# Negative thermal expansion and electronic structure variation of chalcopyrite type LiGaTe_2_†

**DOI:** 10.1039/c8ra01079j

**Published:** 2018-03-12

**Authors:** V. V. Atuchin, Fei Liang, S. Grazhdannikov, L. I. Isaenko, P. G. Krinitsin, M. S. Molokeev, I. P. Prosvirin, Xingxing Jiang, Zheshuai Lin

**Affiliations:** Laboratory of Optical Materials and Structures, Institute of Semiconductor Physics, SB RAS Novosibirsk 630090 Russia atuchin@isp.nsc.ru; Laboratory of Semiconductor and Dielectric Materials, Novosibirsk State University Novosibirsk 630090 Russia; Key Laboratory of Functional Crystals and Laser Technology, Technical Institute of Physics and Chemistry, Chinese Academy of Sciences Beijing 100190 China zlin@mail.ipc.ac.cn; Laboratory of Crystal Growth, Institute of Geology and Mineralogy, SB RAS Novosibirsk 630090 Russia; Laboratory of Functional Materials, Novosibirsk State University Novosibirsk 630090 Russia; Laboratory of Crystal Physics, Kirensky Institute of Physics, Federal Research Center KSC SB RAS Krasnoyarsk 660036 Russia; Department of Physics, Far Eastern State Transport University Khabarovsk 680021 Russia; Surface Science Laboratory, Boreskov Institute of Catalysis, SB RAS Novosibirsk 630090 Russia

## Abstract

The LiGaTe_2_ crystals up to 5 mm in size were grown by the modified Bridgman–Stockbarger technique and the cell parameter dependence on temperature in the range of 303–563 K was evaluated by the X-ray diffraction analysis. The thermal behavior of LiGaTe_2_ is evidently anisotropic and a negative thermal expansion is found along crystallographic direction *c* with coefficient −8.6 × 10^−6^. However, the normal thermal expansion in two *a* directions with coefficient 19.1 × 10^−6^ is dominant providing unit cell volume increase on heating. The atomic mechanism is proposed to describe this pronounced anisotropic expansion effect. The electronic structure of LiGaTe_2_ is measured by X-ray photoelectron spectroscopy and the band structure is obtained by DFT calculations. The pressure response from 0 to 5 GPa was calculated and a normal crystal compression is found. This work indicates that LiGaTe_2_ is promising as an IR NLO or window material for many practical applications because the thermal expansion coefficients of this telluride are not big. We believe that these results would be beneficial for the discovery and exploration of new IR optoelectronic polyfunctional metal tellurides.

## Abstract 2

The LiGaTe_2_ crystals were grown by the Bridgman–Stockbarger technique and the cell parameter dependence on temperature in the range of 303–563 K was evaluated by the X-ray diffraction analysis and first principles calculations.

## Graphical abstract

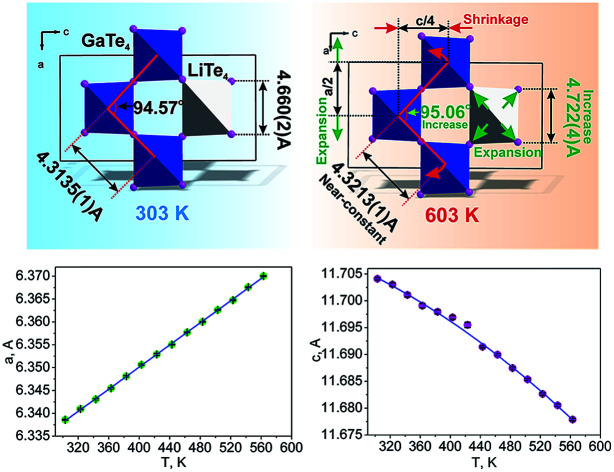

## 1. Introduction

**Fig. 1:**
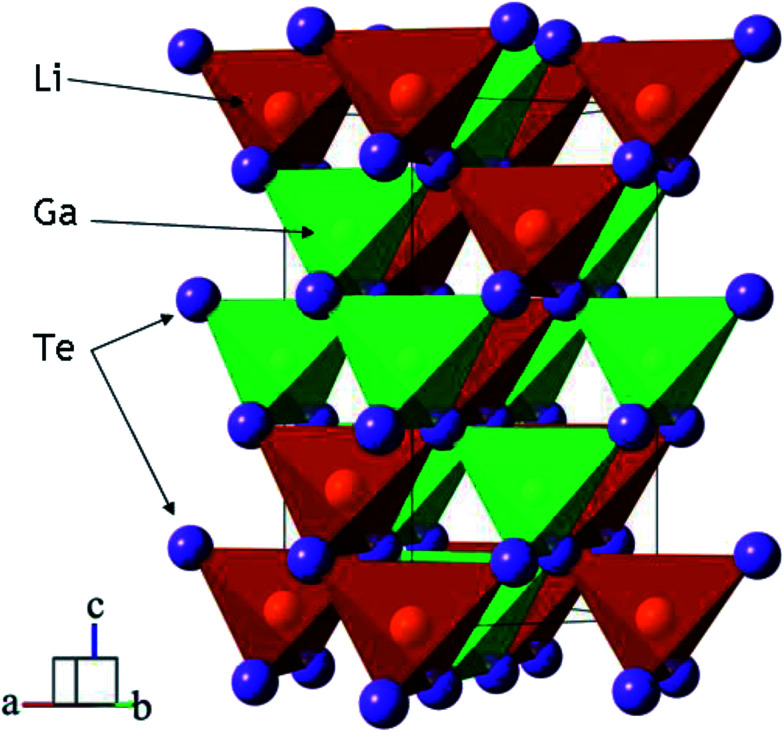
The crystal structure of LiGaTe_2_ chalcopyrite. The unit cell is outlined. Lone lithium and gallium atoms are omitted for clarity.

Telluride crystalline materials are of great importance in modern microelectronics and photonics.^1–12^ Because of specific features of metal-tellurium chemical bonds, tellurides possess narrow-bandgap semiconductor or metallic properties.^1,2,6–8,11,12^ Due to their low-wavenumber phonon spectrum, semiconductor tellurides are transparent in the mid-IR range and they can be used as optical window materials. However, the number of known noncentrosymmetric tellurides is not big and, only for several crystals, the linear and nonlinear properties are obtained.^13–22^ The high potential of LiGaTe_2_ for the optical frequency conversion in the mid-IR spectral range has been demonstrated in several studies, and the structural and optical properties of this material were measured.^13,16,17,20,23^ LiGaTe_2_ is a positive uniaxial crystal and it has a chalcopyrite type structure (*I*4̄3*d* with the closest packing).^24^ The structure of LiGaTe_2_ is shown in Fig. 1.^16,25^ Each Li, Ga and Te atom occupies only one crystallographic position, the Li–Te bond distance is 2.736 Å and, for the Ga–Te bond, the value is 2.611 Å. For comparison, in the LiGaSe_2_ structure (wurtzite type, *Pna*2_1_) there are two positions of Se. The Li–Se bond distance changes from 2.493 to 2.565 Å, while the Ga–Se bonds range from 2.389 to 2.405 Å. The nonlinear crystal LiGaTe_2_ is characterized by the biggest band-gap energy of *E*_g_ = 2.41 eV among tellurides, which is essentially bigger than those of LiInTe_2_ (1.5 eV), AgGaTe_2_ (1.32 eV) and AgInTe_2_ (1.03 eV). A sufficiently large birefringence (0.094) for LiGaTe_2_ allows one to obtain its room-temperature phase-matching in the whole transparency range (1.66–21 μm). Its nonlinear coefficient *d*_36_ was estimated by the phase-matched second harmonic generation to be 43 pm V^−1^, which is about 7 times higher than that of the related orthorhombic LiGaS_2_ and about 4.5 times relative to LiGaSe_2_. The conversion efficiency in LiGaTe_2_ for SHG 10.6 μm was found to be higher than that of AgGaSe_2_.^16^ Besides, this telluride is considered as a promising crystal for neutron detection.^26,27^ However, in several studies, it was reported on that crystal growth of LiGaTe_2_ is not trivial because of its high tellurium volatility and pronounced chemical activity of lithium at high temperatures.^20,28–30^ This may result in the defect generation and stoichiometry deviations, and these effects should be accounted to reach the optical quality crystals.

For a practical NLO crystal, the crystal growth and optical response are equally important. Consequently, the thermal and chemical properties, including electronic structure analysis, play the most crucial role. However, only a few theoretical studies focus on these issues.^31–36^ To date, there are no reports about the dependence of structural parameters of LiGaTe_2_ on temperature and experimental measurements of electronic structure. Thus, the present study is aimed at the LiGaTe_2_ crystals growth and observation of the electronic structure in parallel by the X-ray photoelectron spectroscopy (XPS) and theoretical methods, where XPS is extremely sensitive to chemical state of the crystal surface and theoretical model is a power tool to consider the relations between crystal structure and physical properties. Besides, the dependence of structural parameters of LiGaTe_2_ on temperature is explored combining experimental and theoretical methods.

## 2. Experimental methods

LiGaTe_2_ crystals were obtained in three stages.^37^ At the first stage, we obtained the charge by fusing the elementary components Li, Ga, and Te in a glass carbon crucible inside the sealed quartz ampoule. The ampoule was evacuated to the residual pressure of 10^−3^ atm. The synthesis temperature was 1250 K, the initial components purity was Li 99.99%, for Ga and Te – 99.9999%. All the metal reagents were supplied by Alfa Aesar. The accuracy of reagent mass measurements was ±4 mg. The pyrosynthesis reaction proceeds with the liberation of a large amount of heat. At the conditions, Li reacts with the crucible walls and it is partially consumed. To compensate this effect, we introduced an excess of Li_2_Te to the starting stoichiometric composition at the level up to 16 at%. To avoid the material contamination by lithium oxide and/or nitride, the sample handling was produced in the Ar atmosphere in a specialized dry box. Upon the completion of the active synthesis stage, the melt was maintained at the melting temperature for 24 h.

**Fig. 2:**
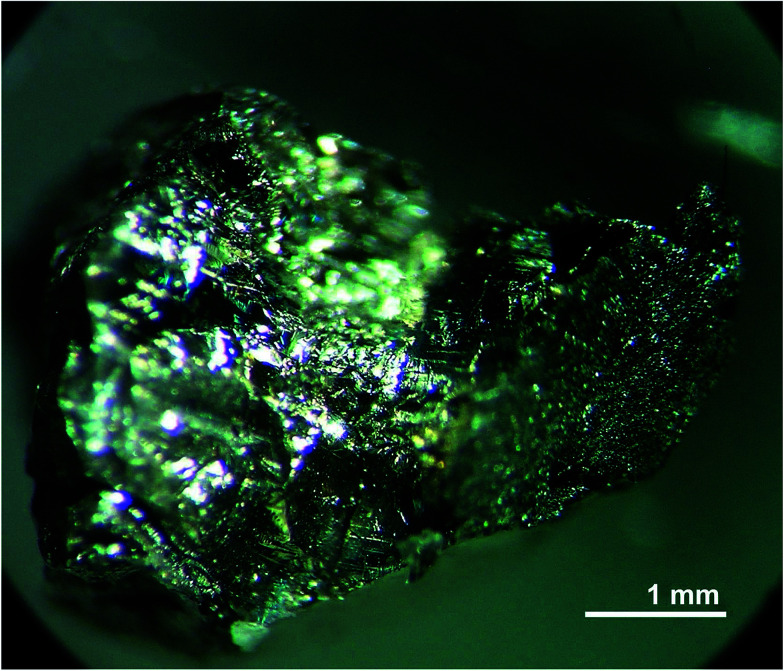
Photo image of the LiGaTe_2_ crystal.

The second stage was the melt homogenization in a sealed quartz ampoule with an inner pyrolytic carbon coating. The homogenization temperature did not exceed 50 K above the melting point of LiGaTe_2_. This level was selected because the incongruent evaporation of the melt appeared above this temperature.^29,30^ The LiGaTe_2_ crystals up to 5 mm in size were grown from the homogenized melt by the Bridgman–Stockbarger technique in the two-zone vertical resistance furnace with the diaphragm in a sealed pyrocarbon inner-coated quartz ampoule. The ampoule was first evacuated to the residual pressure of 10^−3^ atm and then filled with a high purity Ar (*P* = 0.2–0.5 atm). The temperature at the start of crystallization was a few degrees below the LiGaTe_2_ melting point. The maximum gradient near the crystallization front was 2 K mm^−1^ and the ampoule movement rate to the cold zone was 2.5 mm per day. The crystal growth was performed for 20 days. The photo image of the grown crystal is shown in Fig. 2.

The powder diffraction data of LiGaTe_2_ were collected at room temperature with a Bruker D8 ADVANCE powder diffractometer (Cu-Kα radiation) and linear VANTEC detector. The step size of 2*θ* was 0.016° and the counting time was 1.5 s per step. The 2*θ* range of 5–70° was measured with the 0.6 mm divergence slit, but the 2*θ* range of 70–140° was measured with the 2 mm divergence slit. The intensities and obtained esd's were further normalized: *I*_*i* norm_ = *I*_*i*_ × 0.6/(slit width), *σ*_norm_(*I*_*i*_) = *σ*(*I*_*i*_) × 0.6/(slit width), taking into account the actual divergence slit width value which was used to measure each particular intensity *I*_*i*_, and saved in the xye-type file. So, the transformed powder pattern had a usual view over the whole 2*θ* range of 5–140°, but all high-angle points had small esd values.

**Fig. 3:**
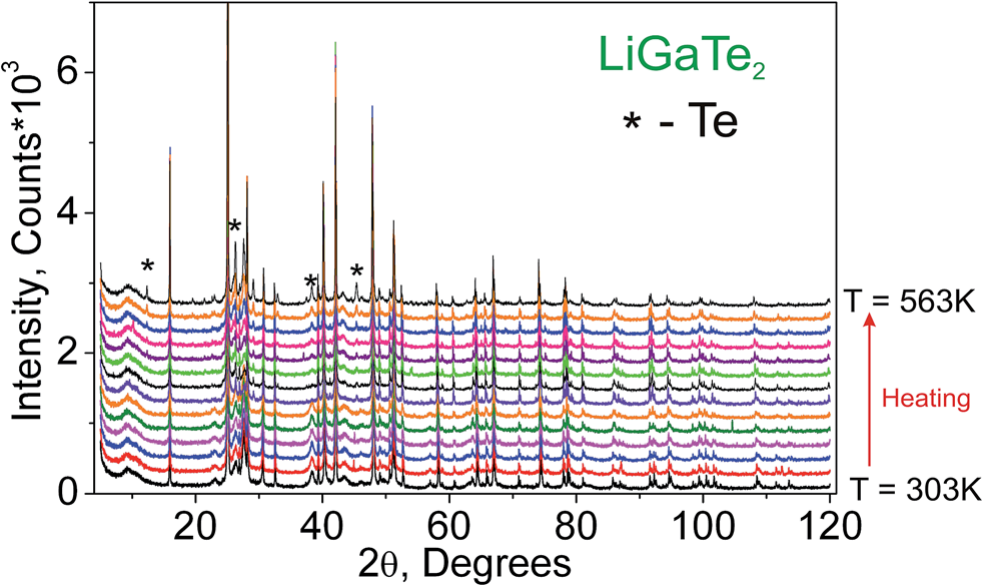
The XRD patterns recorded from the LiGaTe_2_ sample in the range from 303 K to 563 K. The impurity peaks related to the Te component are marked by the asterisk.

Additionally, to see the structural parameters dependence on temperature, fourteen X-ray diffraction (XRD) patterns in the range of 2*θ* = 5–120° were collected at fourteen different temperatures in the range from 303 K to 563 K spending 35 min for each pattern. The measurements were carried out using the temperature set TTK450 (Anton Paar). The recorded XRD patterns are shown in Fig. 3. It was found that the sample decomposes at a low rate in the temperature range of 303–443 K, but, after that, the rate increases and the impurity peaks prevent a good fitting. Therefore, the XRD patterns from 443 K to 563 K were recorded from a new LiGaTe_2_ powder sample. However, the decomposition at 583 K also leads to a large amount of Te impurity, and a further heating leads to the total sample decomposition and we stop the experiment.

The XPS spectra were recorded using a SPECS (Germany) photoelectron spectrometer equipped with a hemispherical PHOIBOS-150-MCD-9 analyzer and FOCUS-500 (Al K_α_ radiation, *hν* = 1486.74 eV, 200 W) monochromator. Just before the measurements, the binding energy (BE) scale was calibrated using the positions of the peaks of Au 4f_7/2_ (BE = 84.0 eV) and Cu 2p_3/2_ (BE = 932.67 eV) core levels. The LiGaTe_2_ powder sample was prepared for the XPS experiment. To decrease a possible contamination by air agents, the LiGaTe_2_ crystal was ground in agate mortar into the glove box filled with high-purity argon. In this way, however, the chemical reaction between LiGaTe_2_ and agate species can not be excluded. The LiGaTe_2_ powder sample was loaded onto a conducting double-sided copper scotch and transfered into the input vacuum chamber without a contact with the air environment. The details of the sample handling and BE energy scale calibration methods can be found elsewhere.^38–40^ The base pressure of a sublimation ion-pumped chamber of the system was less than 5 × 10^−10^ mbar during the present experiments. Besides the survey photoelectron spectra, the narrow spectral regions Li 1s, Te 3d, Ga 2p, Ga 3d, Te 4d and the valence band were recorded. The survey spectra were taken at the analyzer pass energy of 50 eV and the detailed spectra were registered at 20 eV. The concentration ratios of the elements on the sample surface were calculated from the integral photoelectron peak intensities which were corrected with the theoretical sensitivity factors based on Scofield photoionization cross sections.^41^ For the peak fitting procedure, a mixture of Lorentzian and Gaussian functions was used together with the Shirley background subtraction method.

## 3. *Ab initio* calculation methods

The electronic band structures calculations for LiGaTe_2_ were performed by a CASTEP package.^42^ The generalized gradient approximation (GGA-PBE) functionals^43^ were selected to be the exchange–correlation (XC) functional. The ion–electron interactions were described by optimized norm-conserving pseudopotentials.^44^ The orbital electrons of Li 2s^1^, Ga 3d^10^4s^2^4p^1^, Te 5s^2^5p^4^ were treated as valence electrons and the correlation energy (*U* = 5.0 eV) was used to describe the Hubbard U of Ga 3d electrons. The kinetic energy cutoff of 880 eV and Monkhorst–Pack *k*-point meshes^45^ spanning less than 0.04 Å^−3^ (4 × 4 × 2) in the Brillouin zone were chosen to ensure the sufficient accuracy of current methods. The hydrostatic pressure (from 0 to 5 GPa; step 0.5 GPa) was applied on the LiGaTe_2_ crystal and geometry optimization calculations were performed until the energy change, the maximum force and maximum displacement were less than 5.0 × 10^−6^ eV per atom, 0.01 eV Å^−1^, and 5.0 × 10^−4^ Å, respectively. The convergence tests revealed that the above computational parameters are accurate enough for the purposes of this study. The phonon dispersion spectra of LGT were calculated by the linear response method.^46^ Notably, the primitive cell constants under different temperatures were fixed in phonon calculations.

## 4. Results and discussion

### 4.1. Temperature effect

**Fig. 4:**
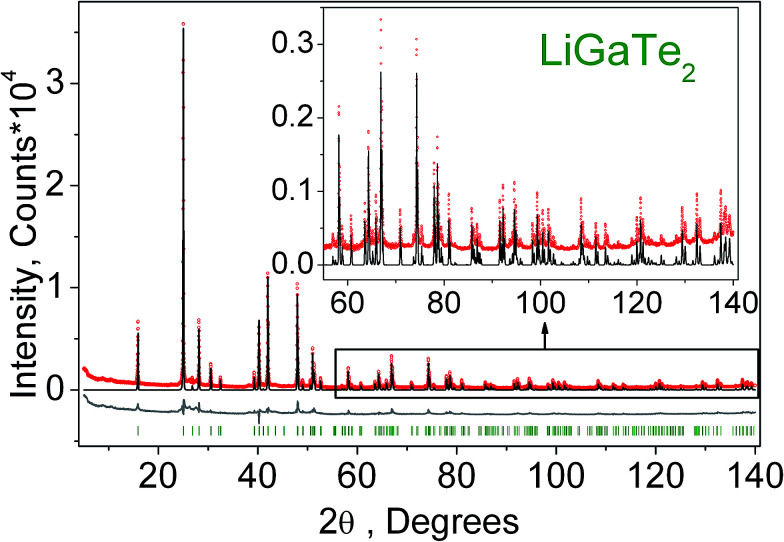
Difference Rietveld plot of LiGaTe_2_.

The crystal structure refinement was performed by the package DDM which accounts the esd values of each point by a special weight scheme.^47^ The powder pattern has a complex background curve due to the existence of an amorphous phase in the sample, and this was the reason for applying the DDM program instead of commonly used Rietveld programs. All diffraction peaks were indexed by the tetragonal cell (*I*4̄2*d*) with parameters close to those of LiGaTe_2_.^16^ Therefore, the crystal structure shown in Fig. 1 was taken as a starting model for the refinement. The thermal parameter of Li was not refined and other ions were refined with isotropic thermal parameters. The refinement was stable and it gave low *R*-factors, as evident from Table 1 and Fig. 4. The atom coordinates and main bond lengths are summarized in Tables S1 and S2,† respectively.

**Table 1:** Main parameters of processing and refinement of the LiGaTe_2_ sample

Compound	LiGaTe_2_
Space group	*I*4̄2*d*
*a*, Å	6.33757 (2)
*c*, Å	11.70095 (5)
*V*, Å^3^	469.966 (4)
*Z*	4
2*θ*-interval, °	5–140
*R*_DDM_, %	9.48
*R*_exp_, %	6.98
*χ* ^2^	1.36
*R*_B_, %	4.74

**Fig. 5:**
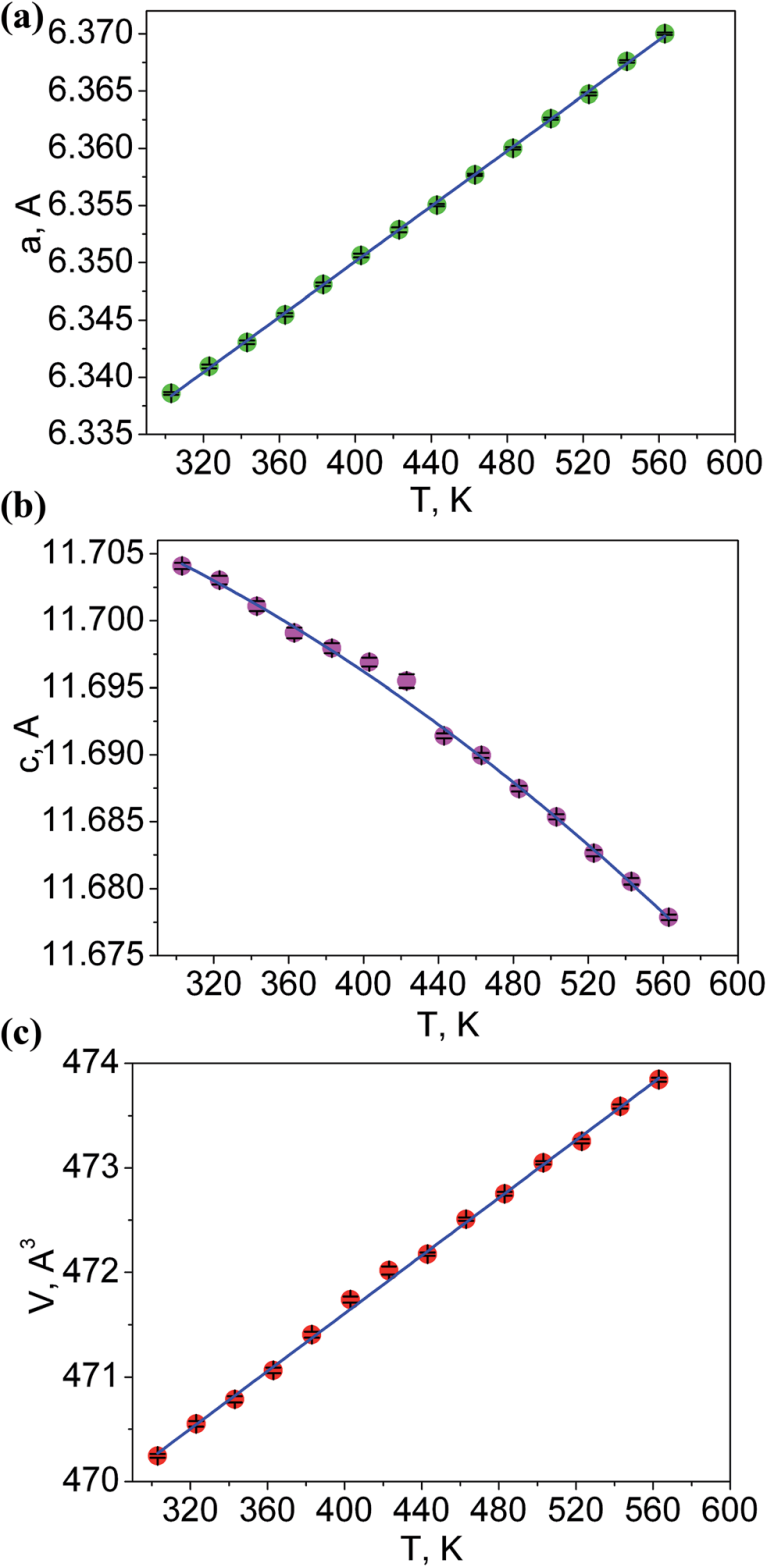
Temperature dependences of cell parameters: (a) *a*; (b) *c*; (c) cell volume *V*.

The additional fourteen powder patterns recorded at the temperatures from 303 K to 563 K were also treated by the DDM program and this gave the thermal dependencies of unit cell parameters and cell volume with a good reliability (Fig. 5, Table S3†). With the temperature increase, one can see a strong increase of *a* cell parameter, but cell parameter *c* noticeably decreases. This means that the thermal behavior of LiGaTe_2_ is evidently anisotropic and the negative thermal expansion is found in crystallographic direction *c*. However, the normal thermal expansion in two *a* directions is dominant; thereby, the cell volume still increases on heating, as shown in Fig. 5c. The thermal expansion coefficients obtained for LiGaTe_2_ are summarized in Table 2. Thus, this telluride should be classified as an anisotropic positive thermal expansion (APTE) material.

**Table 2:** Thermal expansion coefficients in LiGaTe_2_

Crystallographic parameter	Thermal expansion coefficient, K^−1^
*a*	19.1 × 10^−6^
*c*	−8.6 × 10^−6^
*V*	29.4 × 10^−6^

**Fig. 6:**
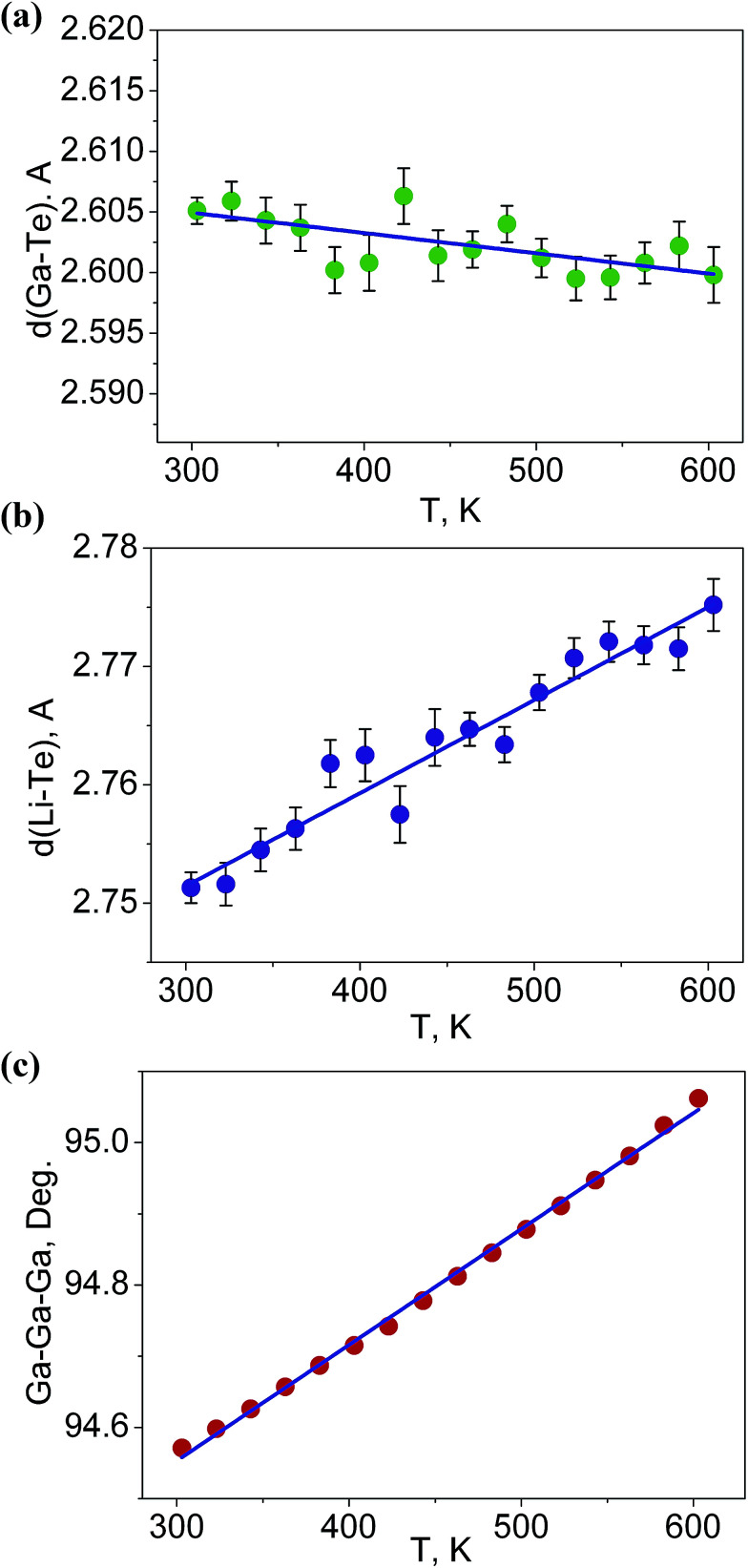
The dependences of (a) *d*(Ga–Te), (b) *d*(Li–Te) bond lengths and (c) Ga–Ga–Ga angle on temperature.

**Fig. 7:**
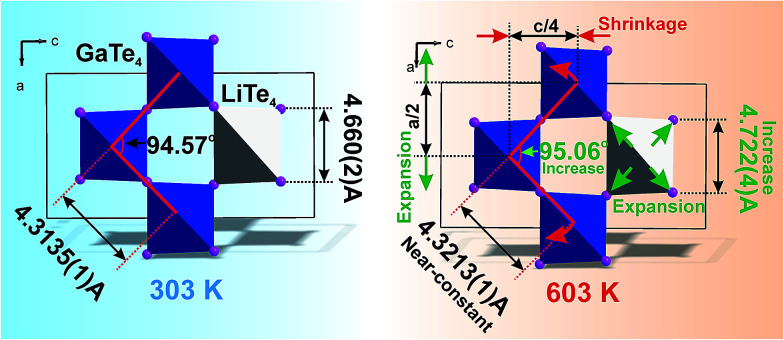
The model which explained the increase of *a* cell parameter and the decrease of *c* cell parameter in LiGaTe_2_ on heating. The bond length *d*(Li–Te) increases, but *d*(Ga–Te) lengths stay almost unchangeable under heating. This leads to the asymmetrical deformation of the (GaTe_4_)_3_(LiTe_4_) ring and the increase of Ga–Ga–Ga angle which is responsible for the expansion of parameter *a* and the shrinkage of the parameter *c*.

It is interesting to consider the atomic mechanism of this pronounced anisotropic expansion effect. The dependences of *d*(Ga–Te) and *d*(Li–Te) bond lengths on temperature are shown in Fig. 6. Accordingly, as the temperature increase from 303 to 563 K, the Ga–Te bond length fluctuation is only 0.2% (Fig. 6a). This implies that the Ga–Te bond length is almost persistent, manifesting the rigidity of GaTe_4_ tetrahedra when being heated. In comparison, the Li–Te bond length exhibits a higher flexibility and it increases from 2.751 to 2.775 Å (by 0.9%, almost five times than that of the Ga–Te bond, Fig. 6b). Coupled with the Li–Te bond elongation, the neighboring rigid GaTe_4_ tetrahedra rotate with each other, leading to the enlargement of the Ga–Ga–Ga angle from 94.57° to 95.06° (Fig. 6c). This effect directly results in the increase of the projection along the a-axis and the contraction of that along *c*-axis in the Ga–Ga distance with the increasing temperature, giving rise to the positive and negative thermal expansions of these two dimensions, respectively. Therefore, the APTE behavior of LiGaTe_2_ is mainly ascribed to the synergetic effect between the configuration modification of “rigid” GeTe_4_ and “flexible” LiTe_4_ tetrahedra, as depicted in Fig. 7.

As it is known, the thermal expansion response is strongly related to intrinsic phonon vibrational modes. In order to elucidate the mechanism of negative thermal expansion along the *c* axis, we performed the phonon calculations of LGT at different temperatures (303, 403, 503 and 603 K) (Table 3). The lattice parameters are fixed and the atomic positions are fully relaxed in calculations. The relationship between the Grüneisen parameter and thermal expansion coefficient is given as:
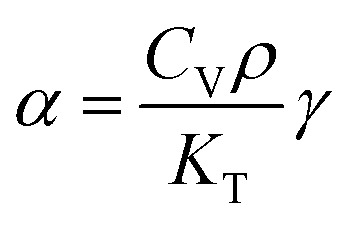
where *α* is the thermal expansion coefficient, *C*_v_ is the principle heat capacity, *ρ* is density, *K*_T_ is isothermal bulk moduli, *γ* is the macroscopic Grüneisen parameter. The macroscopic Grüneisen parameter *γ* is the sum of the microscopic Grüneisen parameters (*γ*_*i*_) of the phonon model by weight. And then, the microscopic Grüneisen parameter of an individual vibrational mode with number *i* can be defined as (the negative of) the logarithmic derivative of the corresponding frequency *ω*_*i*_:
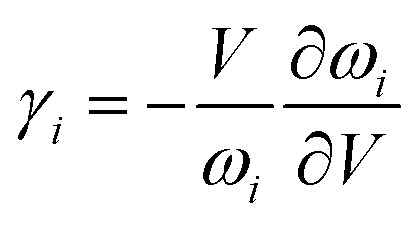
and *V* is the volume. So, if the frequency of a vibrational mode increases with the increasing volume (increasing temperature), this phonon mode would contribute to the negative thermal expansion (NTE).

**Table 3:** The detailed atomic vibrations (cm^−1^) for the optical modes by the first-principles calculations

Mode	303 K	403 K	503 K	603 K
E	**54.099**	**54.249**	**54.379**	**54.346**
E	**54.099**	**54.249**	**54.379**	**54.346**
B_2_	**75.147**	**75.198**	**75.308**	**75.321**
E	**79.036**	**79.157**	**79.263**	**79.381**
E	**79.036**	**79.157**	**79.263**	**79.381**
B_1_	**84.277**	**84.360**	**84.508**	**84.643**
A_2_	94.289	93.849	93.444	93.083
A_2_	117.890	117.494	117.150	116.817
A_1_	139.510	139.153	138.849	138.488
B_2_	186.916	186.231	185.674	185.160
B_1_	192.383	191.624	191.000	190.352
E	199.834	199.356	198.992	198.668
E	199.834	199.356	198.992	198.668
E	208.451	208.116	207.867	207.655
E	208.451	208.116	207.867	207.655
B_2_	300.283	297.476	294.839	292.215
E	306.310	303.520	300.971	298.416
E	306.310	303.520	300.971	298.416
E	311.169	308.399	305.858	303.364
E	311.169	308.399	305.858	303.364
B_1_	312.970	310.324	307.852	305.353

For a chalcopyrite structure with space group *I*4̄2*d*, symmetry allows the optical vibrations to be classified into 15 normal modes: Γ = A_1_+ 2A_2_ + 3B_1_ + 3B_2_ + 6E, where B_2_ and E are polar Raman active modes having transverse optical (TO) and longitudinal optical (LO) modes, A_1_ and B_1_ are nonpolar Raman active modes, and A_2_ is an optically inactive mode. As seen in Table 3, six vibrational modes (low frequency region) exhibit an anomalous wavenumber increase on the increasing temperature. So, we can assigned the negative thermal expansion of LGT to these vibration modes. As shown in Fig. S1,† the B_2_ modes located at 75.14 cm^−1^ (300 K) mainly contribute to the rotation of (GaTe_4_) tetrahedra, coupling the Li^+^ slide migration along the *c* axis. Therefore, the negative thermal expansion of LGT along the *c* axis can be attributed to the tetrahedra rotation, combining the experimental measurements and theoretical calculations.^48^

### 4.2. Comparison between XPS spectra and band structure

The survey photoemission spectrum recorded from the LiGaTe_2_ sample is shown in Fig. S2.† All detected spectral features were successfully attributed to constituent element core levels and Auger lines, except for the weak C 1s and O 1s bands shown in Fig. S3 and S4,† respectively. A comparatively narrow dominant component of the C 1s line (284.8 eV) was related to adventitious hydrocarbons adsorbed at the surface from the air, and the low-intensity additive component at 289.7 eV was attributed to carbonate species. In the O 1s line, two components can be revealed. The lower binding energy component at ∼530.4 could be attributed to the surface oxide, and a higher binding energy component appeared at ∼531.5 eV could be related to the initial stages of surface hydration. Thus, it can be concluded that LiGaTe_2_ is not chemically inert and it actively interacts with atmospheric agents. From the technological point of view, this should be accounted for at crystal cutting and polishing stages.

**Fig. 8:**
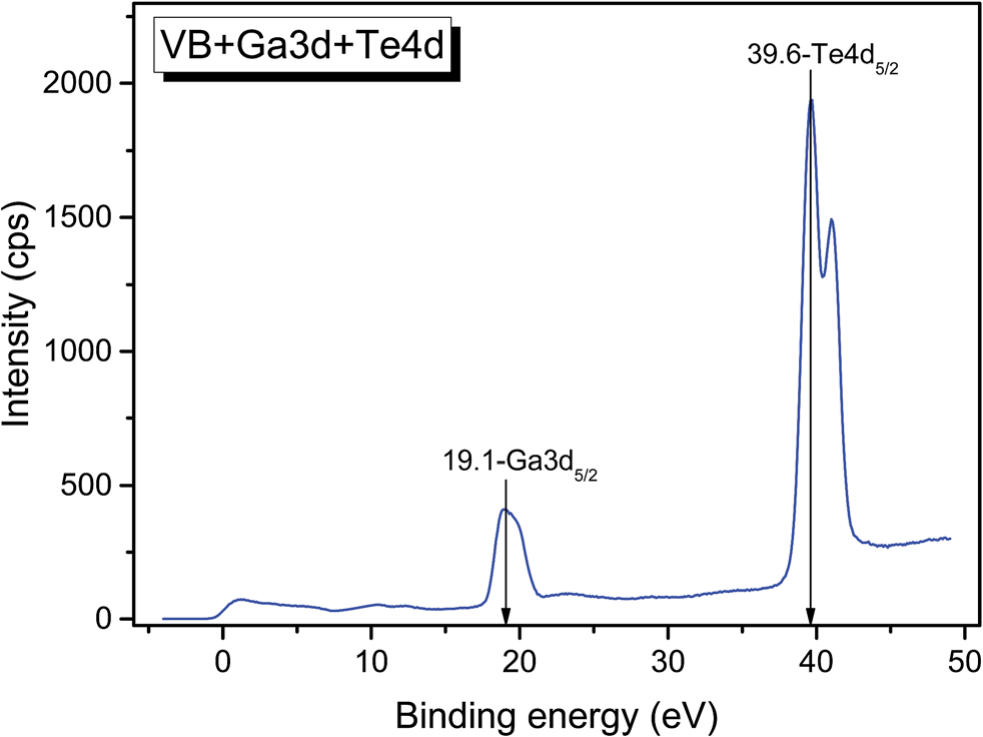
Detailed XPS spectrum of the Ga 3d and Te 4d doublets from LiGaTe_2_.

**Fig. 9:**
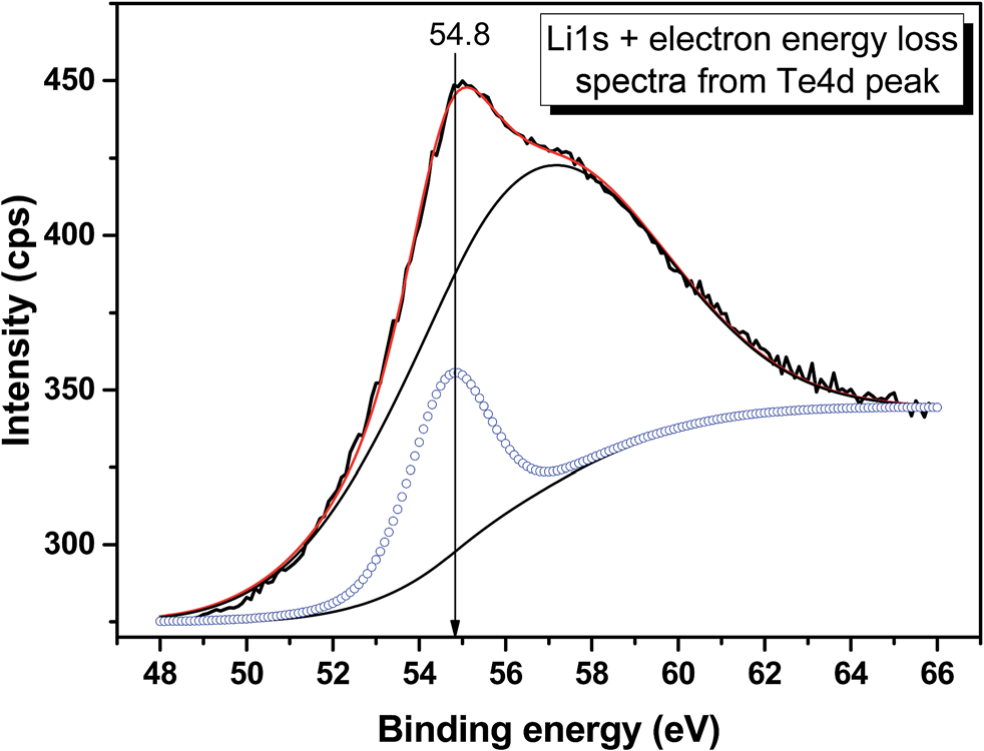
Detailed XPS spectrum of the Li 1s core level from LiGaTe_2_.

**Fig. 10:**
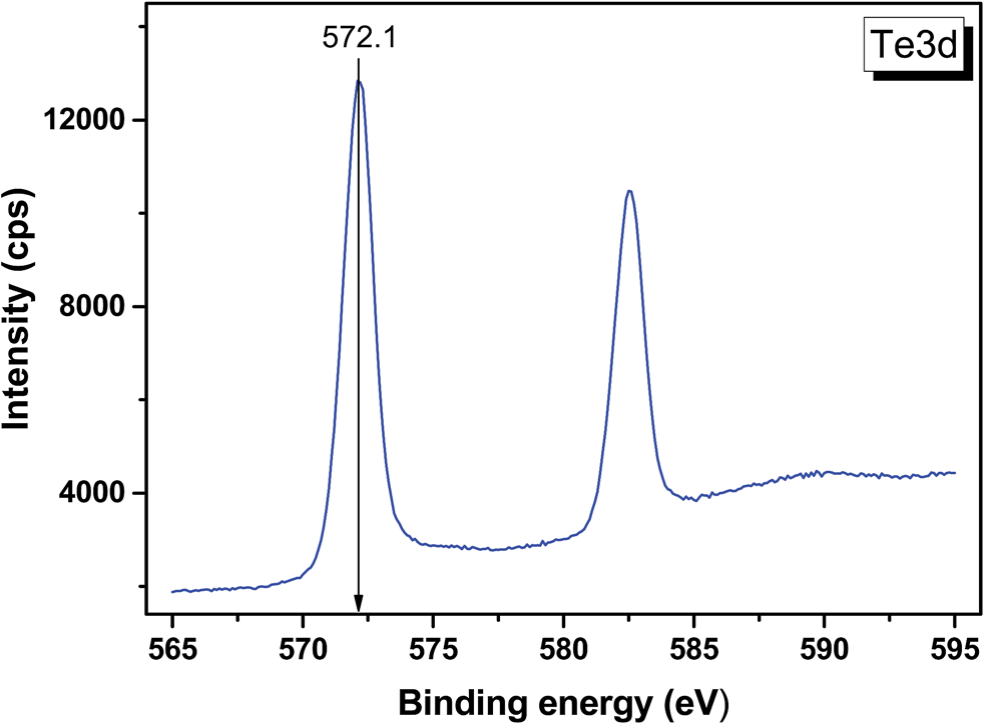
Detailed XPS spectrum of the Te 3d doublet from LiGaTe_2_.

**Fig. 11:**
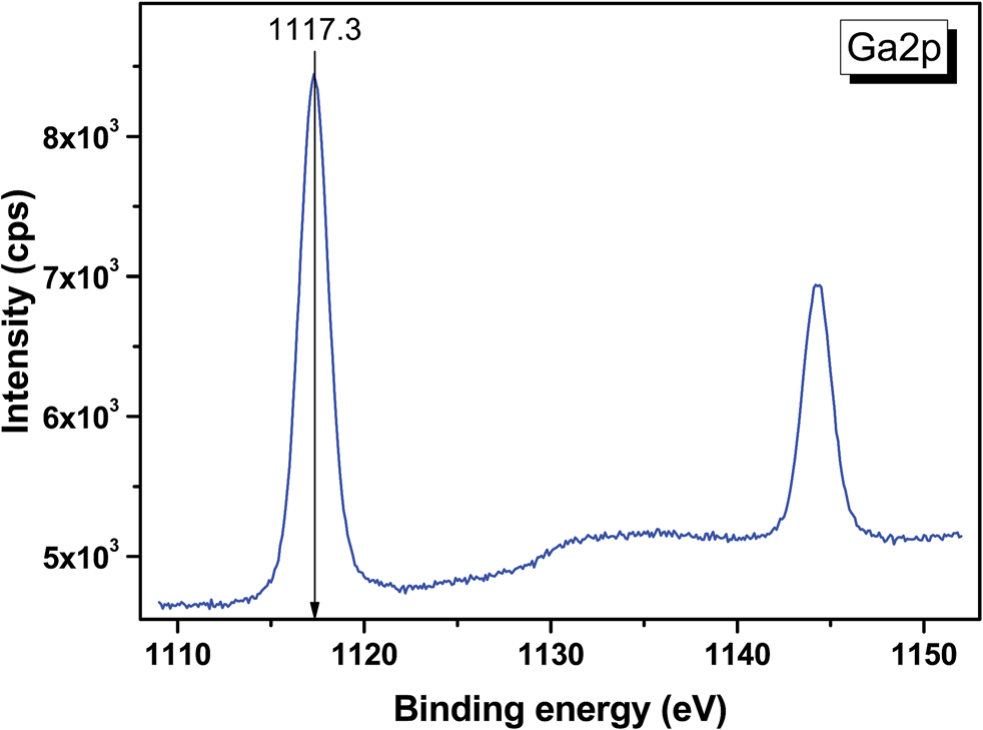
Detailed XPS spectrum of the Ga 2p doublet from LiGaTe_2_.

The valence band spectrum and the nearest constituent element core levels are shown in Fig. 8. The mixed-states bands with several less pronounced components are found over the binding energy range of 0–17 eV. At ∼19.5 and ∼40 eV, the bands related to Ga 3d and Te 4d are observed, respectively. In Fig. 9–11, the representative element core levels Li 1s, Te 3d and Ga 2p are shown. The Li : Ga : Te ratio was estimated by Li 1s, Te 4d and Ga 3d peak areas and tabulated atomic sensitivity factors.^41^ The relative element ratio for the powder sample is Li : Ga : Te = 0.38 : 0.22 : 0.40 and that indicates a noticeable enrichment of the LiGaTe_2_ surface by Li. Previously, a similar effect was observed for closely related compound LiGaS_2_.^49^ Thus, the Li segregation to the crystal surface may be a general feature of compounds LiGaX_2_ (X = S, Se, Te). The ratio Ga : Te = 0.55 is in reasonable consistence with nominal composition Ga : Te = 0.50. The calculations were made without carbon signal accounting. The set of the element core levels measured for LiGaTe_2_ is shown in Table 4. Comparatively, the BE values obtained for Li 1s, Te 3d and Ga 2p core levels in LiGaTe_2_ are in a good relation with the BE values obtained earlier in several other representative compounds.^49–52^

**Table 4:** Binding energies of the constituent element core levels in LiGaTe_2_

Core level	Binding energy, eV				
	LiGaTe_2_	LiGaS_2_	GaTe	PbTe(100)	Bi_2_Te_3_(001)
Ga 3d	19.1	19.7	19.5	—	—
Te 4d_5/2_	39.6	—	—	—	—
Te 4d_3/2_	41.0	—	—	—	—
Li 1s	54.8	54.9	—	—	—
C 1s	Fixed at 284.8, 289.7	Fixed at 284.6, 288.4	—	—	Fixed at 284.8
O 1s	531.5	531.5	530.8	530.0	—
Te 3d_5/2_	572.1	—	573.1	571.9	572.5
Te 3d_3/2_	582.5	—	583.5	—	582.8
Ga 2p_3/2_	1117.3	1117.6	1117.9	—	—
Ga 2p_1/2_	1144.2	114.5	—	—	—
Reference	This study	49	50	51	52

**Fig. 12:**
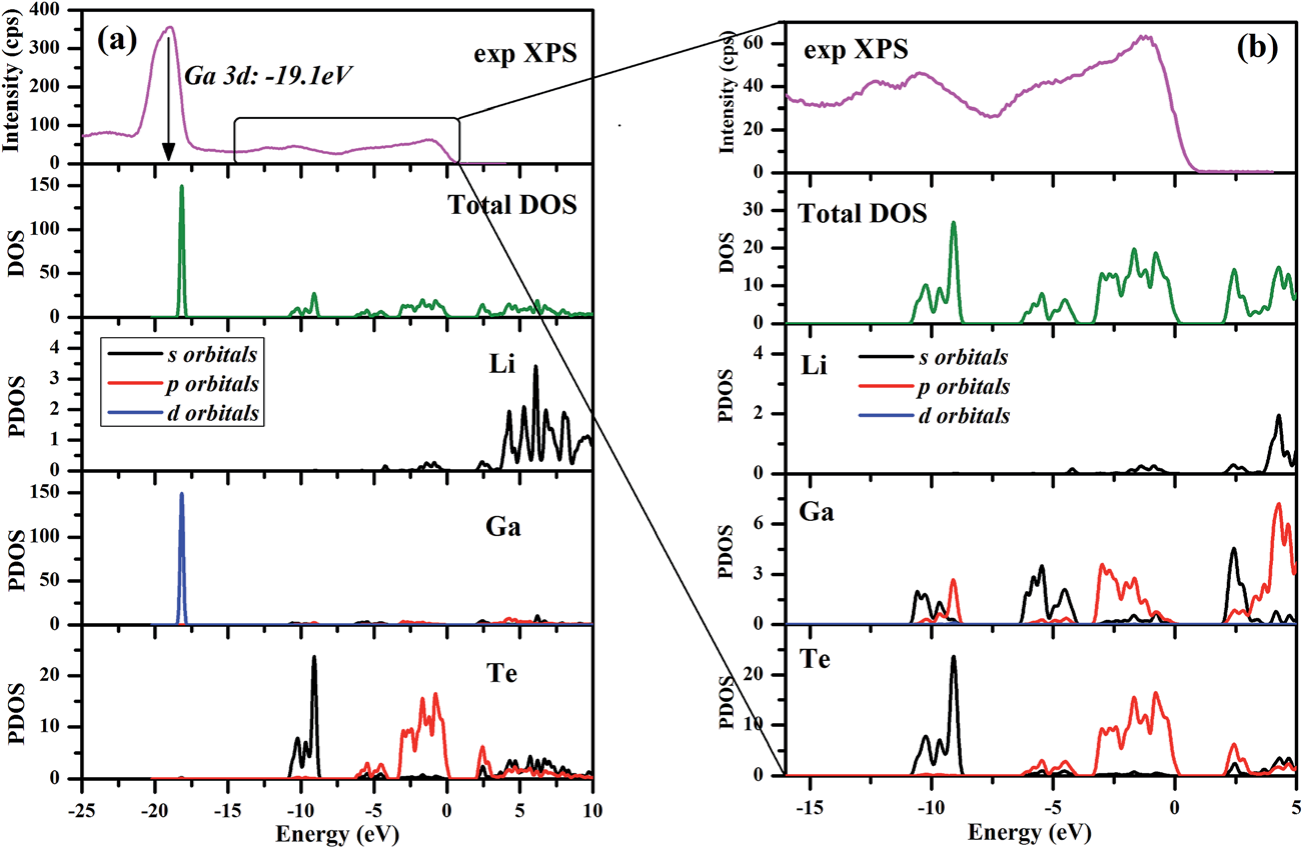
Electronic total and partial densities of states (DOS and PDOS) in LiGaTe_2_.

The calculated electronic total and partial densities of states (DOS and PDOS) projected on the constituent elements in LiGaTe_2_ are displayed in Fig. 12. LiGaTe_2_ is non-magnetic compound, so it spin-up and spin-down band structure (and DOS) are degenerated. Therefore, we only plotted one spin tunnel component for clarity. It is noted that the Te 4d orbitals cannot be simulated by DFT because they are inner-shell electron levels and there are no suitable pseudopotentials that take them into account. As displayed in Fig. 12a, the XPS spectrum and electron density of states are reasonably matched in the energy region from −20 to 0 eV. This confirms the validity of the plane-wave pseudopotential method in its application to LiGaTe_2_. Some bond characteristics can be deduced from the PDOS: (i) the Ga 3d orbitals are strongly localized at −19.1 eV, which is consistent with the calculated strong DOS peak at −18.16 eV. It hybridizes little with the orbitals of other elements, suggesting that Ga 3d electrons are not bonded with Te orbitals. Notably, the Hubbard U values mainly changes the position of Ga 3d orbitals (from −15.2 eV at *U* = 0 eV to −18.16 eV at *U* = 5 eV); (ii) the Te 5s orbitals are relatively localized at the inner energy level around −10 eV, which is slightly hybridized with Ga 4s and 4p electrons; (iii) from 10 to 0 eV, the electron states are mainly composed of Ga 4s/4p and Te 5s/5p orbitals. The strong hybridization in the wide energy range between these orbitals indicates the strong covalent interaction between Ga and Te atoms. From −10 to −8 eV, Te 5s orbitals play a crucial role. Meanwhile, Ga 4s and Te 5p orbitals make the main contribution to the hybridization interaction. Notably, the valence band maximum is mainly composed of Ga 3p and Te 5p orbitals. In addition, Li 1s orbitals are only located at the valence band maximum with a negligible contribution; (iv) the bottom of the conduction band minimum is mainly contributed from the Ga 4s and Te 5p orbitals. Since the optical effects of a crystal are mainly determined by the optical transition between the electronic states close to the bandgap, it is anticipated that they are dominantly contributed from [GaTe_4_] tetrahedra, and the contribution from the orbitals of Li^+^ cations is negligibly small.

### 4.3. Optical properties

LiGaTe_2_ is a direct gap crystal with the calculated bandgap (1.85 eV) (Fig. S5†), which is slightly smaller than the experimental value (2.30 eV) owing to the well-known band gap underestimation by the GGA exchange–correlation (XC) functional.^53^ Therefore, a scissors operator^54^ is usually introduced to shift all the conduction bands in order to agree to the experimental band gap value for calculating the optical coefficients. The calculated second order NLO coefficient is *d*_36_ = −52.7 pm V^−1^, which is comparable to other calculated results.^55^ If adopting GGA+U method, the calculated *d*_36_ is −49.6 pm V^−1^. The Hubbard U mainly changes the position of Ga 3d orbitals but no influence on the electric bands near forbidden gap (see Fig. S6†). Because the optical effects of a crystal are mainly determined by the optical transition between the electronic states close to the band gap, accordingly, it is anticipated that the effects of Hubbard U are negligibly small on optical properties. In addition, the birefringence is calculated to be 0.035 (see Fig. S7†), which is smaller than the experimental results,^16^ but it agrees well to other calculated results.^34,55^

### 4.4. Pressure effect

**Fig. 13:**
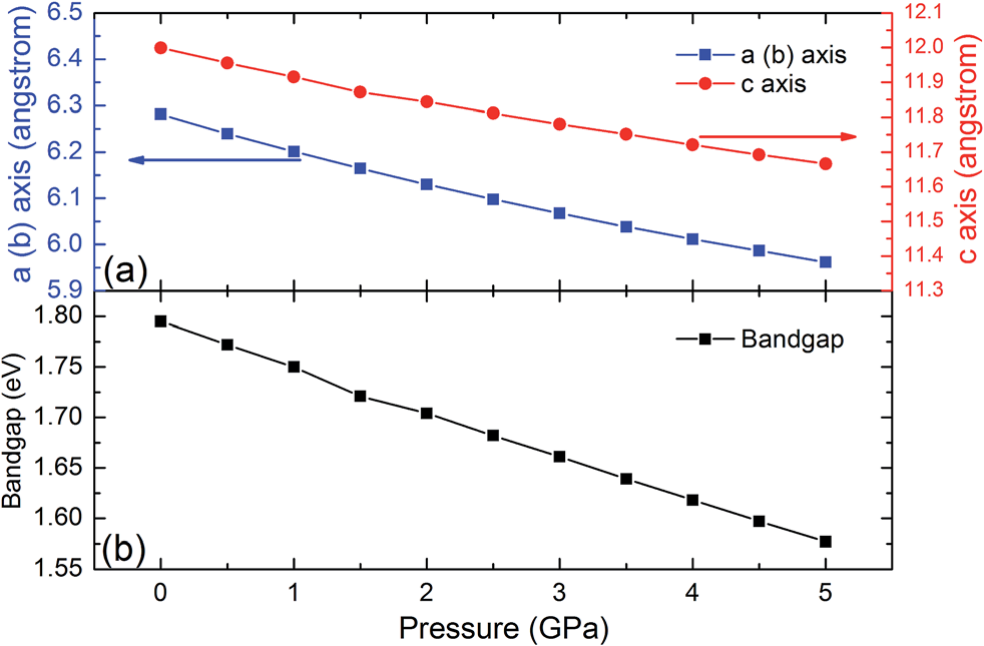
The crystal lattice constants (*a* and *c*) and band gap energy of LiGaTe_2_ as a function of pressure.

The SHG coefficients of NLO materials are highly dependent on the band gap energy. Band gap engineering is generally achieved through lattice distortion by the impurity doping that results in changing the material composition. Since this method often leads to some negative change that could not be controlled well, material compression under high pressure is a remarkable alternative for changing the lattice parameters while keeping the element composition. To identify the high pressure effects on the properties of the LiGaTe_2_ crystal, the lattice constants and band gap energy dependence on the pressure applied uniformly is also investigated. The crystal lattice constants (*a* and *c* axis) and the band gap energy of the LiGaTe_2_ at different pressures are shown in Fig. 13. The *a* and *c* axes compression up to 5% and 3%, respectively, refers to the pressure uniformly applied on the crystal in the range of 0–5 GPa. The LiGaTe_2_ band gap decreases monotonically with the increasing pressure, which is similar to that of Li_2_S^56^ and As_2_S_3_.^57^ For the As_2_S_3_ crystal, the optical band gap red-shifts rapidly with the increasing pressure, decreasing from 2.7 eV at the ambient condition to 1.6 eV at 10 GPa. Owing to a positive correlation between the band gap and laser induced damage threshold (LIDT), it indicates that the LIDT value of LiGaTe_2_ will decrease with applying the external pressure.

## 5. Conclusions

In this study, the big-sized high-quality LiGaTe_2_ crystal was grown by the Bridgman–Stockbarger method. The anisotropic thermal expansion behavior of the LiGaTe_2_ crystal was measured for the first time. Combining the experimental characterization and theoretical calculations, the negative thermal expansion along the *c* axis was assigned to the rotational vibration mode of (GaTe_4_) tetrahedra. The negative thermal expansion was first found in chalcopyrite-type chalcogenides and, respectively, searching for similar structural effects in other materials from this wide crystal family is topical. In addition, the electronic structure of LiGaTe_2_ crystal was measured by XPS and the recorded valence band is in a good agreement with the theoretical electronic density of states. Moreover, the optical properties and pressure response from 0 to 5 GPa were also calculated in the theory. These results indicate that LiGaTe_2_, besides its well known pronounced linear and nonlinear optical properties in IR spectral range, possesses the specific structural effects that may be a way to new applications of this telluride material.

## Conflicts of interest

There are no conflicts to declare.

## Supplementary Material

RA-008-C8RA01079J-s001
