# Supplementary material for: Negative thermal expansion and electronic structure variation of chalcopyrite type LiGaTe2
Source: RSC Adv. 2018 Mar 12;8(18):9946–55. doi: 10.1039/c8ra01079j (PMC9078859; doi:10.1039/c8ra01079j)
Supplement: RA-008-C8RA01079J-s001 [file RA-008-C8RA01079J-s001.pdf]

Electronic Supplementary Material (ESI) for RSC Advances.
This journal is © The Royal Society of Chemistry 2018

Supplementary Materials

Negative thermal expansion and electronic structure variation of chalcopyrite type $LiGaTe_2$

V.V. Atuchin[1,2], Fei Liang[3], S. Grazhdannikov[4,5], L.I. Isaenko[4,5], P.G. Krinitsin[4,5], M.S. Molokeev[5,6,7], I.P. Prosvirin[8], Xingxing Jiang[3], Zheshuai Lin[3]

[1]Laboratory of Optical Materials and Structures, Institute of Semiconductor Physics, SB RAS, Novosibirsk 630090, Russia

[2]Laboratory of Semiconductor and Dielectric Materials, Novosibirsk State University, Novosibirsk 630090, Russia

[3] Key Laboratory of Functional Crystals and Laser Technology, Technical Institute of Physics and Chemistry, Chinese Academy of Sciences, Beijing 100190, China

[4]Laboratory of Crystal Growth, Institute of Geology and Mineralogy, SB RAS, Novosibirsk 630090, Russia

[5]Laboratory of Functional Materials, Novosibirsk State University, Novosibirsk 630090, Russia

[6]Laboratory of Crystal Physics, Kirensky Institute of Physics, Federal Research Center KSC SB RAS, Krasnoyarsk 660036, Russia

[7]Department of Physics, Far Eastern State Transport University, Khabarovsk 680021, Russia

[8]Surface Science Laboratory, Boreskov Institute of Catalysis, SB RAS, Novosibirsk 630090, Russia

Table S1. Fractional atomic coordinates and isotropic displacement parameters (Å^2) of $LiGaTe_2$

	x	y	z	U_{iso}
Te	0.26834 (18)	3/4	1/8	0.0138 (5)
Ga	0	0	1/2	0.0201 (11)
Li	0	0	0	0.0126

Table S2. Main bond lengths (Å) of $LiGaTe_2$

Ga—Te^{i}	2.6086 (7)	Li—Te^{ii}	2.7462 (7)

Symmetry codes: (i) -*y*+1/2, *x*-1/2, -*z*+1/2; (ii) *x*, *y*-1, *z*.

Table S3. Main parameters of refinement of the $LiGaTe_2$ sample from 303 to 583 K

T, K	Space group	Cell parameters (°, Å), Cell volume (Å^3)	R_{DDM}, R_B (%), χ^2
303	*I*-42*d*	*a* = 6.33859 (11),<br>*c* = 11.7040 (2),<br>*V* = 470.24 (2)	14.64, 6.7, 1.14
323	*I*-42*d*	*a* = 6.34095 (16),<br>*c* = 11.7030 (3),<br>*V* = 470.55 (3)	15.24, 7.85, 1.21
343	*I*-42*d*	*a* = 6.34306 (16),<br>*c* = 11.7011 (4),<br>*V* = 470.79 (3)	15.30, 7.90, 1.16
363	*I*-42*d*	*a* = 6.34547 (15),<br>*c* = 11.6991 (4),<br>*V* = 471.06 (3)	15.60, 7.79, 1.16
383	*I*-42*d*	*a* = 6.34710 (16),<br>*c* = 11.6979 (4),<br>*V* = 471.41 (3)	15.60, 8.21, 1.12
403	*I*-42*d*	*a* = 6.35063 (16),<br>*c* = 11.6969 (3),<br>*V* = 471.74 (3)	16.17, 7.21, 1.15
423	*I*-42*d*	*a* = 6.3529 (2),<br>*c* = 11.6955 (5),<br>*V* = 472.02 (4)	15.64, 8.57, 1.10
443	*I*-42*d*	*a* = 6.3550 (1),<br>*c* = 11.6914 (2),<br>*V* = 472.17 (2)	13.52, 8.17, 1.18
463	*I*-42*d*	*a* = 6.35769 (9),<br>*c* = 11.6900 (2),<br>*V* = 472.51 (2)	14.25, 7.73, 1.20
483	*I*-42*d*	*a* = 6.36001 (11),<br>*c* = 11.6875 (2),<br>*V* = 472.75 (2)	13.86, 8.10, 1.20
503	*I*-42*d*	*a* = 6.3626 (1),<br>*c* = 11.6854 (2),<br>*V* = 473.05 (2)	14.00, 8.37, 1.19
523	*I*-42*d*	*a* = 6.36473 (12),<br>*c* = 11.6826 (2),<br>*V* = 473.26 (2)	14.84, 7.99, 1.22
543	*I*-42*d*	*a* = 6.36758 (11),<br>*c* = 11.6805 (2),<br>*V* = 473.59 (2)	14.86, 9.01, 1.21
563	*I*-42*d*	*a* = 6.3700 (1),<br>*c* = 11.6779 (2),	15.69, 8.35, 1.23

$V = 473.85$ (2)

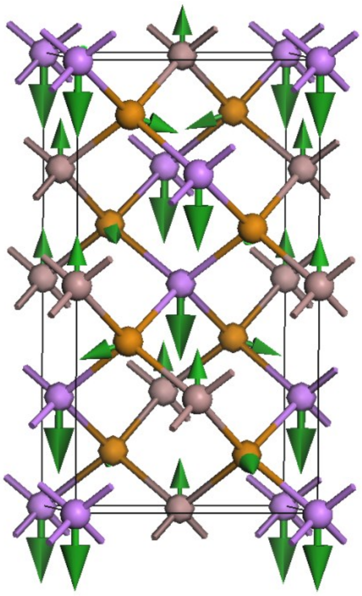

Figure S1. The diagram of vibrational mode of 75.14 cm^{-1}.

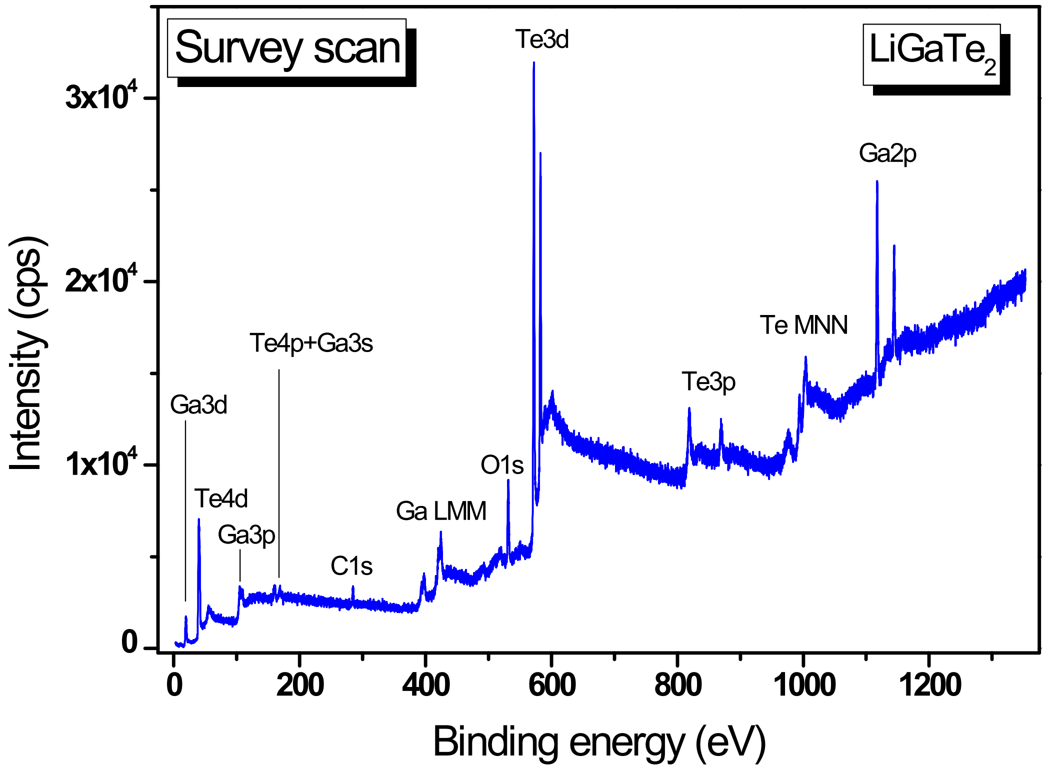

Figure S2. Survey photoelectron spectrum of $LiGaTe_2$.

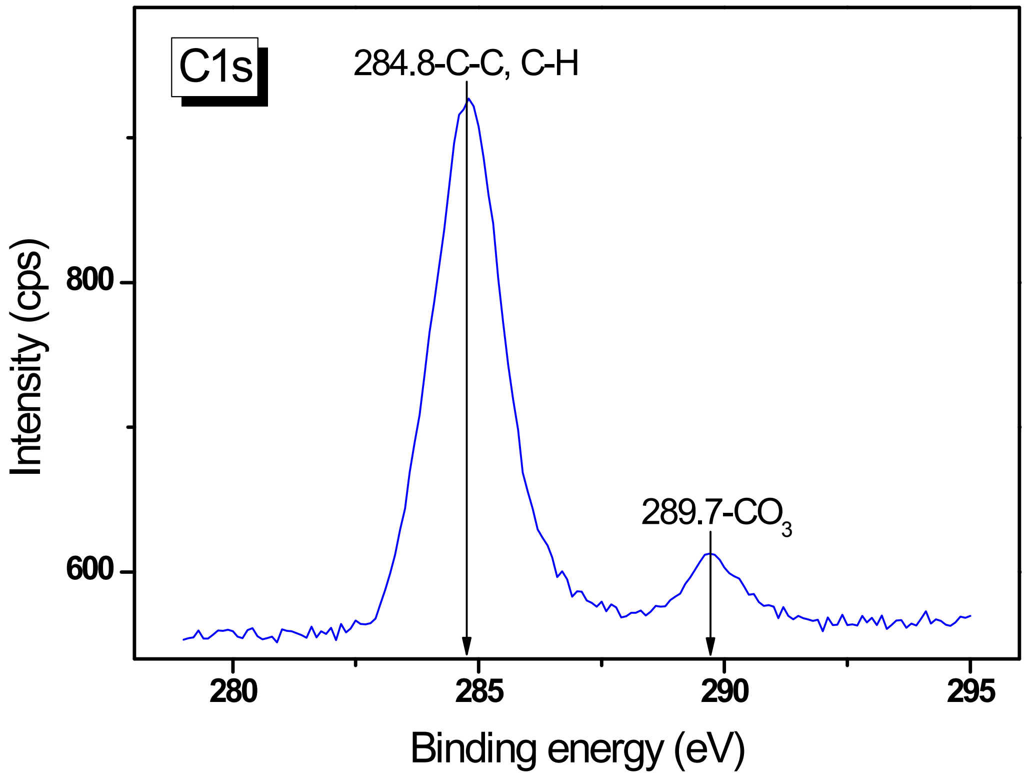

Figure S3. C 1s core level.

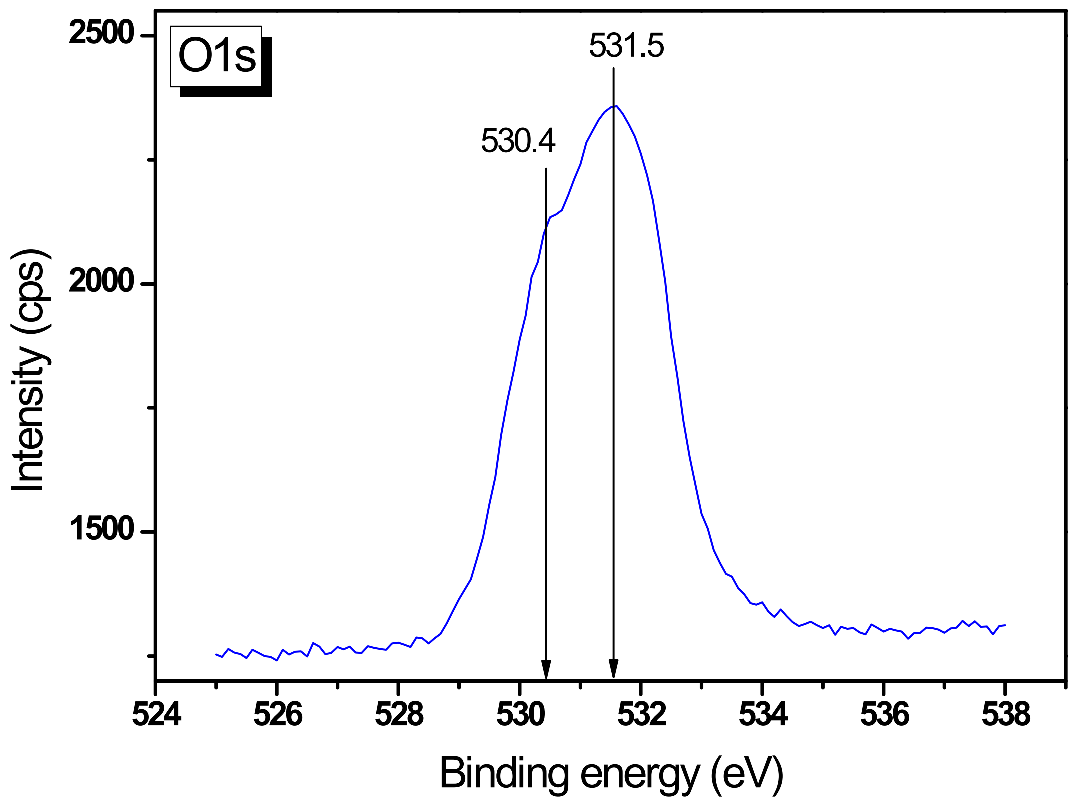

Figure S4. O 1s band.

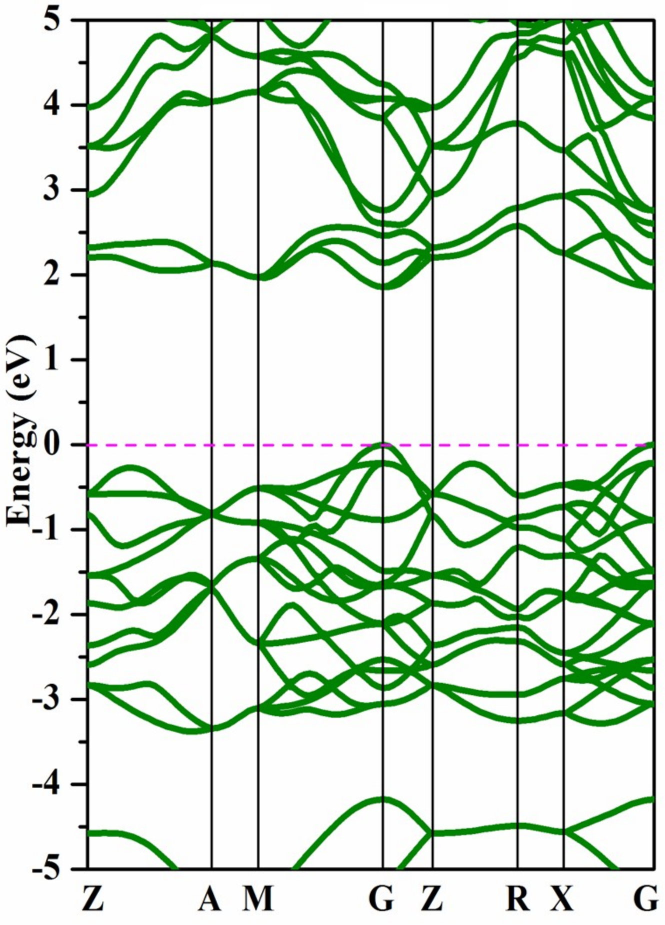

Figure S5. Electronic band structure of $LiGaTe_2$, calculated by PBE functional.

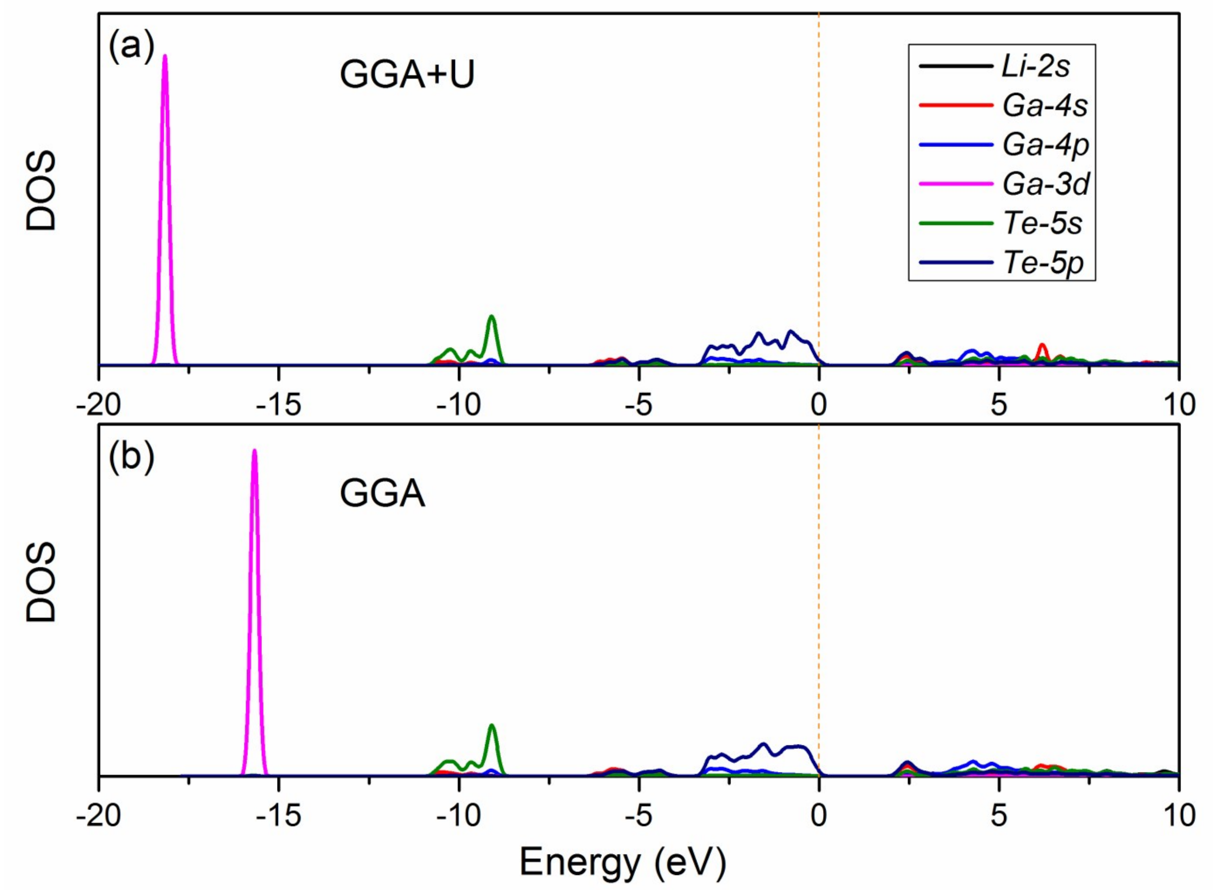

Figure S6. the calculated density of states of $LiGaTe_2$. (a) GGA+U, (b) GGA

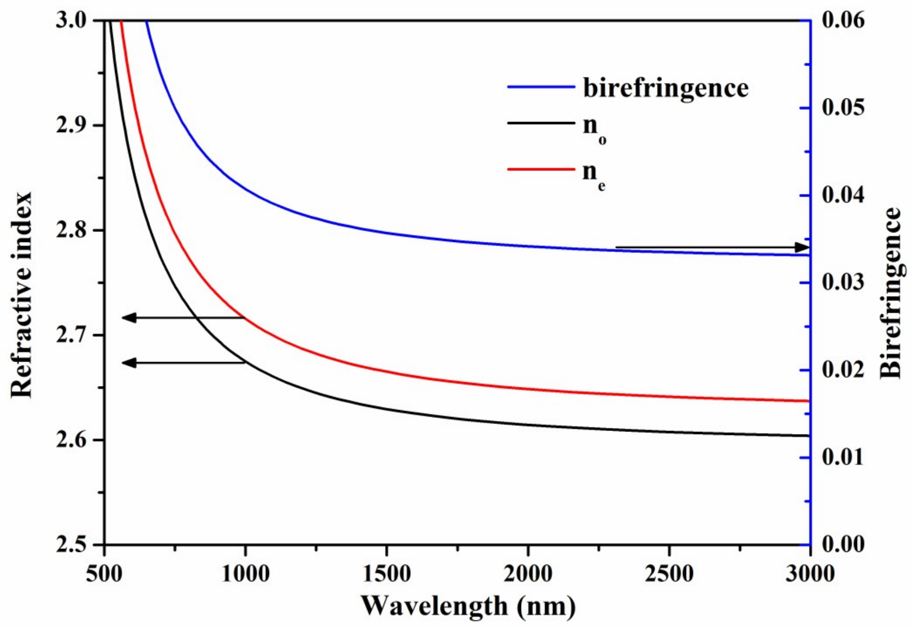

Figure S7. The calculated refractive indexes and birefringence of $LiGaTe_2$ crystal.
